# LXRɑ participates in the mTOR/S6K1/SREBP-1c signaling pathway during sodium palmitate-induced lipogenesis in HepG2 cells

**DOI:** 10.1186/s12986-018-0268-9

**Published:** 2018-05-02

**Authors:** Youping Zhou, Shengjie Yu, Can Cai, Li Zhong, Huihong Yu, Wei Shen

**Affiliations:** 1 Department of Gastroenterology, 2nd Affiliated Hospital of Chongqing Medical University, Chongqing, 400010 China; 2 Department of Urology, 2nd Affiliated Hospital of Chongqing Medical University, Chongqing, 400010 China

**Keywords:** LXRα, mTOR, S6K1, NAFLD, HepG2 cells

## Abstract

**Background:**

The aim of this study was to investigate how the signaling pathway downstream of mTOR/S6K1 contributes to the regulation of SREBP-1c expression during lipogenesis in HepG2 cells.

**Methods:**

The model of steatosis was established using human hepatocytes HepG2 and inducting them with sodium palmitate. mTOR, S6K1 and LXRα were inhibited by rapamycin, PF-4708671 and siRNA-LXRα, respectively. After a variety of different treatment, the levels of intracellular triglycerides, the accumulation of lipid droplets and the expression levels of related genes were detected.

**Results:**

Rapamycin, PF-4708671 and siRNA-LXRα treatment could decrease the accumulation of triglycerides and lipid droplets induced by sodium palmitate in HepG2 cells, and the inhibitory effect could be enhanced by the combination of them. Sodium palmitate stimulated the expression of genes encoding mTOR, S6K1, LXRα, SREBP-1c and SREBP-1c target enzymes (FAS and ACC1) in HepG2 cells. Moreover, these genes were sensitive to rapamycin. PF-4708671 also decreased the expression of these genes, except for the mTOR gene, and the extent of reduction could be enhanced by combination with rapamycin. Knockdown of LXRα decreased the expression of SREBP-1c, FAS and ACC1, but it had no effect on the expression of mTOR or S6K1. Furthermore, rapamycin and PF-4708671 enhanced the inhibitory effect of siRNA-LXRα.

**Conclusions:**

mTOR/S6K1 regulates the SREBP-1c signaling pathway through LXRα in sodium palmitate-induced HepG2 cells, suggesting LXRα might be a potential therapeutic target for NAFLD.

## Background

Non-alcoholic fatty liver disease (NAFLD) is a common chronic liver disease worldwide. NAFLD is clinically defined by the accumulation of excess lipids, mainly in the form of triacylglycerol, exceeding 5-10% of the liver weight, in the absence of any primary cause (such as significant alcohol intake, viral hepatic infections, or other specific causes of liver disease) [1]. It has been reported that approximately 25.24% of the global population and 7.6% of children are affected with NAFLD [2, 3]. Patients with NAFLD tend to suffer from metabolic syndromes including obesity, type 2 diabetes mellitus and dyslipidemia [4].These data highlight the global importance of understanding NAFLD pathogenesis, which remains incompletely understood.

NAFLD pathogenesis is considered to be associated with the mammalian target of rapamycin (mTOR) signaling pathway [5].NAFLD is a manifestation of systemic insulin resistance, and mTOR and S6K1 are key components of the insulin signaling pathway [6, 7]. mTOR is a serine/threonine protein kinase that acts as an important molecular junction between nutrients and metabolic processes indispensable for cell growth. Moreover, mTOR is known for its effect on cellular metabolism and is able to regulate endoplasmic reticulum (ER) stress and autophagy inhibition [8]. S6 kinase 1 (S6K1) is a downstream effector of the PI3K/Akt/mTOR pathway, and its kinase activity regulates subsequent lipogenic gene expression induced by SREBP-1c [9, 10]. Sterol regulatory element-binding protein-1c (SREBP-1c) belongs to the nuclear transcription factor family that can activate the transcription of multiple genes encoding for enzymes involved in synthesis of total cholesterol (TC), triglycerides (TGs), fatty acids (FAs) and phospholipids, such as acetyl-CoA carboxylase (ACC)-1 and fatty acid synthase (FAS) [11].

However, the role of the mTOR/S6K1 pathway in the regulation of SREBP-1c expression in the liver has not yet been determined. The regulation of SREBP-1c expression has been reported to be both independent of and dependent on S6K1 levels [9, 12]. Moreover, it has been shown that S6K1 contributes to liver X receptor (LXR)-α activation [13]. LXRα belongs to nuclear receptors that are ligand-activated transcription factors regulating the expression of several genes through the direct modulation of transcriptional activities and epigenetic changes. In humans, hepatic LXR expression has been shown to increase with the increasing severity of NAFLD [14]. LXRα plays a dual role in NAFLD. SREBP-1c is a target of LXRα because the promoter of the SREBP-1c gene contains an LXR reaction element (LXRE) [15]. Hence, we speculate that SREBP-1c and LXRα are correlated with each other, but future studies are needed to investigate the associated regulatory mechanisms. We believe that better understanding of the role of the mTOR pathway in NAFLD would benefit the discovery of an effective therapy for NAFLD.

## Methods

### Cell culture and reagents

HepG2 cells, a human hepatocellular carcinoma cell line that was obtained from the Cell Bank of the Institute of Biochemistry and Cell Biology, were cultured in Dulbecco’s modified Eagle’s medium(DMEM) supplemented with 10% fetal bovine serum (FBS) and antibiotics (100 units/mL penicillin and 100 μg/mL streptomycin) at 37 °C in a humidified incubator containing 5% CO2. Saturated FA (sodium palmitate) was prepared with bovine serum albumin (BSA) solution as previously described [16]. Rapamycin (RAPA) was obtained from Alexis Biochemicals and PF-4708671 was obtained from Selleck Chemicals (USA); both agents were dissolved in dimethyl sulfoxide (DMSO). Unless otherwise stated, cells were treated with 144 μM sodium palmitate, 14 nM RAPA or 6 μM PF-4708671.

Transient small interfering RNA (siRNA) transfection.

Human-specific LXRα-targeting small interfering RNA (siRNA-LXRα) was purchased from GenePharma Biotechnology Co. (China). The sequence of the siRNA-LXRα and scrambled siRNA were 5’-GCAACTCAATGATGCCGAGTT-3′ and 5’-GTTCTCCGAACGTGTCACGT-3′, respectively. HepG2 cells at 70-80% confluence were transfected with either siRNA-LXRα or scrambled siRNA using siRNA Transfection Reagent siTran 1.0 (OriGene Technologies, USA) according to the manufacturer’s instructions. The transfection efficiency was measured by counting EGFP-positive cells using a fluorescence microscope. The medium was replaced 3-6 h after transfection. After transfection for 48 h, cells were collected, and the siRNA-mediated silencing effect was detected by Western blot analysis as described below. If necessary, 24 h after transfection, cells were treated with the indicated concentrations of sodium palmitate for an additional 24 h and harvested for the subsequent processing.

### Oil red O staining

After treatment with sodium palmitate as described above, cells were washed twice in phosphate-buffered saline (PBS) and fixed with 4% formaldehyde for 30 min. After being washed twice in PBS, cells were stained for 15 min in freshly diluted Oil Red O solution at room temperature. The cells were then washed once with 60% isopropyl alcohol and twice with water. Cell nuclei were counterstained with hematoxylin. Representative photomicrographs were captured at 200X magnification using a system incorporated in the microscope. The Oil Red O staining images were analyzed using Image-Pro® Plus 6.0 (Media Cybernetics Corp., Rockville, Maryland, USA). Three representative fields were randomly selected from each group to calculate the Integrated Optical Density (IOD) value of the lipid droplets, and the intracellular lipid content in each group was compared.

### Quantification of intracellular TGs

Intracellular TG levels were measured using commercial kits (Applygen Technologies Inc., China) according to the manufacturer’s instructions. Briefly, after treatment, cells were washed twice with PBS and lysed with RIPA buffer for 10 min at room temperature. The supernatant was transferred into a new tube and incubated for 10 min at 70 °C. These mixtures were centrifuged for 5 min at 2000 rpm. Then, the supernatant was used for enzymatic determination. The data were summarized as the means±standard deviations (SDs).

### RNA isolation and quantitative real-time PCR (qRT-PCR)

Total RNA was extracted from cultured HepG2 cells using TRIzol reagent (Invitrogen, USA), and cDNA was synthesized using a RevertAid First Strand cDNA Synthesis kit (Fermentas, Canada) according to the manufacturer’s recommendations. qRT-PCR was performed using SYBR Premix Ex Taq™ Kit (TaKaRa, Japan). All primers were designed and synthesized by GenePharma Biotechnology Co.(China).The primer sequences for amplifying mTOR, S6K1, LXRα, SREBP-1c, FAS, ACC1 and β-actin were as follows: mTOR (forward 5’-AGCATCGGATGCTTAGGAGTGG-3′ and reverse 5’-CAGCCAGTCATCTTTGGAGACC-3′); S6K1 (forward 5’-TATTGGCAGCCCACGAACACCT-3′ and reverse 5’-GTCACATCCATCTGCTCTATGCC-3′); LXRα (forward 5’-TGGGAACACGATGGGAGAAC-3′ and reverse 5’-CTGCCGTGCCACCTTG-3′); SREBP-1c (forward 5’-ACACAGCAACCAGAAACTCAAG-3′ and reverse 5’-AGTGTGTCCTCCACCTCAGTCT-3′); FAS (forward 5’-TTCTACGGCTCCACGCTCTTCC-3′ and reverse 5’-GAAGAGTCTTCGTCAGCCAGGA-3′); ACC1 (forward5’-TTCACTCCACCTTGTCAGCGGA. -3′ and reverse 5’-GTCAGAGAAGCAGCCCATCACT-3′); and β-actin (forward5’-TGACGGTCAGGTCAT. CACTATCGG-3′ and reverse 5’-TTTGATCTTCATGGTGATAGGAGCGA-3′). β-actin cDNA served as an internal control. All reactions were performed in triplicate. The change in the expression of the target genes (mTOR, S6K1, LXRα, SREBP-1c, FAS and ACC1) were calculated relative to the mean critical threshold (CT) values of the β-actin gene.

### Western blotting analysis

Western blotting analysis was performed as previously described [16]. Briefly, the samples of protein extracted from cells were separated by 5-15% sodium dodecyl sulfate-polyacrylamide gel electrophoresis (SDS-PAGE) and subsequently transferred to a polyvinylidene difluoride (PVDF) membrane. After blocking with PBST containing 5% fat-free milk or 5% BSA (Sigma, USA) for 1 h, the membranes were incubated overnight at 4 °C with specific primary antibodies against phospho-mTOR (Ser2448) (1:1000; Abcam), mTOR (1:1000; Abcam), phospho-S6K1 (Thr389) (1:1000; Cell Signaling Technology), S6K1(1:1000; Abcam), LXRα (1:1000; Abcam), SREBP-1c (1:500; Santa Cruz Biotechnology), FAS (1:200; Santa Cruz Biotechnology), ACC1 (1:1000; Abcam) or GAPDH (1:1000; Sigma), followed by incubation with horseradish peroxidase-linked secondary antibodies at room temperature for 1 h. Protein bands were visualized using enhanced chemiluminescent reagents (Millipore, USA). The band intensity of immunoblots was quantified with Quantity One software (Bio-Rad Laboratories, Hercules, CA).

### Detection of cell viability

Cell viability was assessed using the Cell Counting Kit-8 (CCK-8). Cells were seeded into 96-well plates at 1 × 10^4^ cells/well in 100 μL medium and treated as indicated. After that, the medium was replaced with fresh medium containing the CCK solution (10 μL), and the plate was then incubated at 37 °C for 2 h. The absorbance of each well was detected at 450 nm with an enzyme-linked immunosorbent assay plate reader.

### Statistical analysis

Data are expressed as the means±SDs. All experiments were performed in triplicate. Differences among groups were determined using the Student-Newman-Keuls test. Probability (P)-value < 0.05 was considered statistically significant.

## Results

### Sodium palmitate increases the accumulation of TGs and lipid droplets

**Fig. 1:**
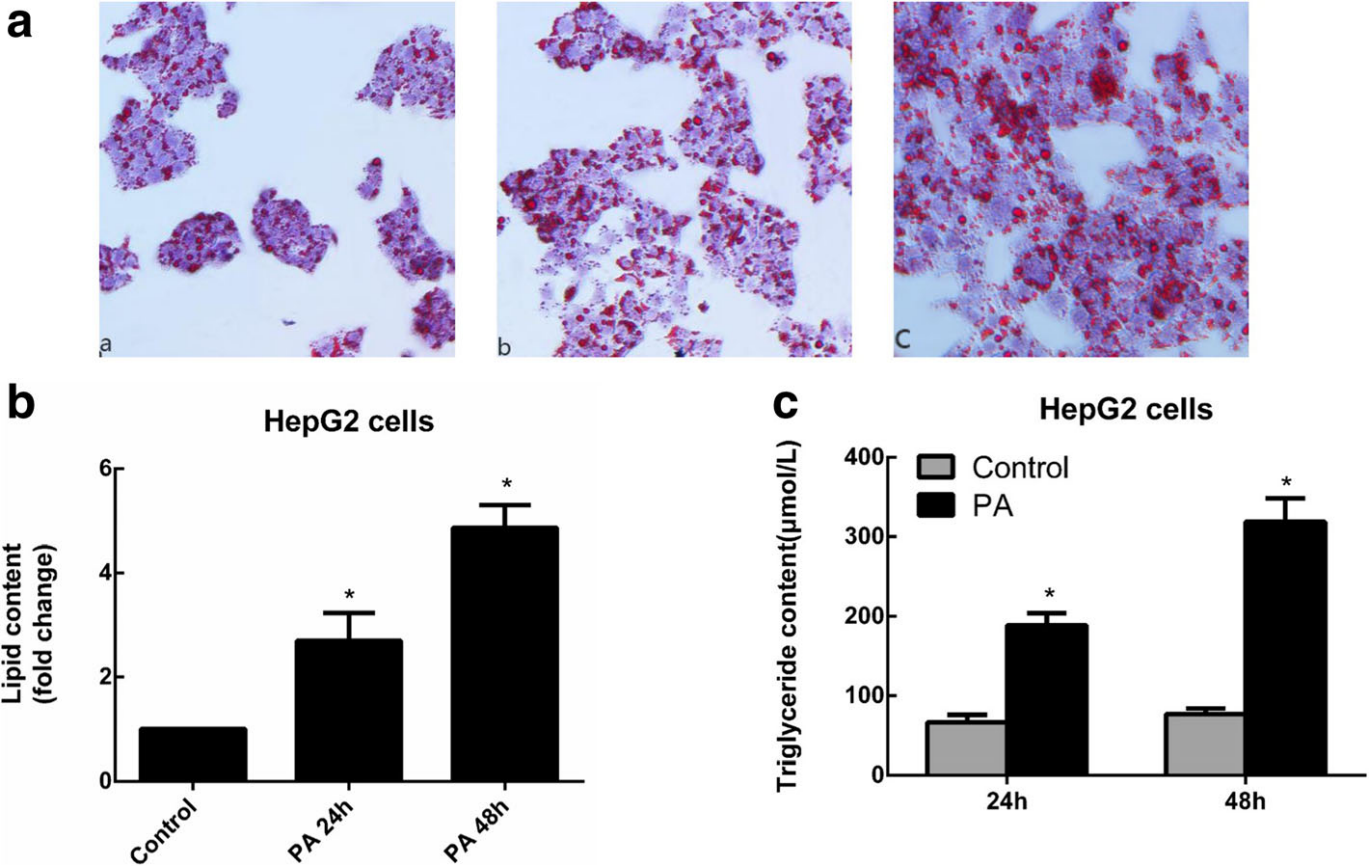
Oil Red O staining and TG content in sodium palmitate-treated HepG2 cells. **a** After incubation with 144 μM PA for different periods of time (a control; b PA for 24 h; and c PA for 48 h), HepG2 cells were stained with Oil Red O and reviewed under an optical microscope. **b** The intracellular lipid content in each group was quantified. **c** Cells were treated with 144 μM PA for 24 and 48 h, and TG levels were measured by an enzymatic assay kit. Data are presented as the means±SDs (*P < 0.05 versus the control group)

Saturated free fatty acids (FFAs), such as PA, have lipotoxicity as documented in many other previous study. In this study, the model of steatosis was established using human hepatocytes HepG2 and inducting them with a low cytotoxic dose of 144 μM sodium palmitate (PA). After treatment for 24 h and 48 h, compared to those in the control HepG2 cells, the accumulation of lipid droplets was significantly increased (Fig. 1a and b) and the levels of intracellular TGs were increased in a time-dependent manner in sodium palmitate-treated HepG2 cells (188.8 ± 15.43 μmol/L and 318.9 ± 29.3 μmol/L, respectively; Fig. 1c). Thus, we were able to successfully establish a steatosis model using HepG2 cells.

### Sodium palmitate-induced lipid accumulation is associated with the increase in both mTOR/S6K1 and LXRα levels via the upregulation of downstream lipogenic genes

**Fig. 2:**
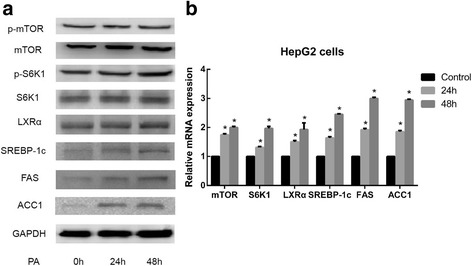
Effect of sodium palmitate on the expression of mTOR/S6K1, LXRα and lipogenic genes in HepG2 cells. Cells were treated with PA as indicated for 24 h and 48 h. Under these conditions, the protein levels of mTOR, S6K1, LXRα, SREBP-1c, FAS, ACC1 and phosphorylated mTOR, S6K1 were determined by Western blotting (**a**), and mRNA expression levels of lipogenesis-related markers were determined by qRT-PCR (**b**). Data are presented as the means±SDs of three independent experiments, each of which was performed in triplicate (*P < 0.05 versus the control group)

To elucidate the mechanism of action of sodium palmitate, the protein and mRNA expression levels of SREBP-1c, a transcription factor that controls lipogenesis, and its target enzymes (FAS and ACC1) were examined using Western blotting and qRT-PCR, respectively. Previous studies have demonstrated that SREBP-1c activation and lipogenesis requires the mTOR/S6K1 pathway [17] and that SREBP-1c is a target of LXRα. Hence, the expression of mTOR, S6K1 and LXRα was also detected. As shown in Fig. 2a and b, the protein expression levels of mTOR, S6K1, LXRα, SREBP-1c, FAS, ACC1 and phosphorylated mTOR, S6K1, and mRNA expression levels of lipogenesis-related markers were were significantly upregulated in a time-dependent manner in the sodium palmitate-treated hepatocytes. Therefore, sodium palmitate-induced lipid accumulation is associated with the increase in both mTOR/S6K1 and LXRα levels via the upregulation of downstream lipogenic genes.

### RAPA suppresses sodium palmitate-induced lipid accumulation and lipogenesis in HepG2 cells by decreasing the expression of S6K1, LXRα and lipogenic genes

**Fig. 3:**
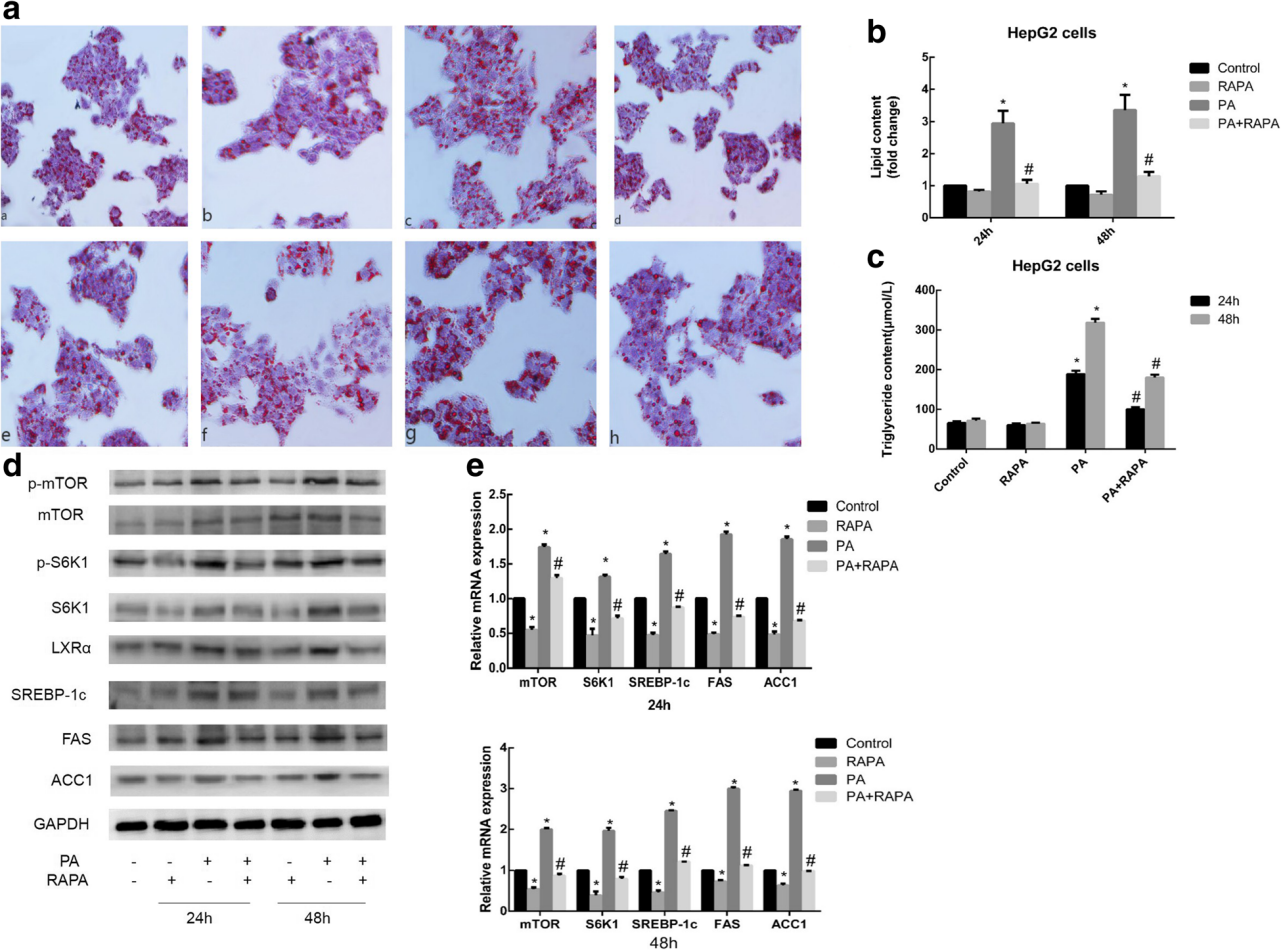
Effect of RAPA on lipid accumulation and expression of S6K1, LXRα and lipogenic genes in HepG2 cells. **a** Cells were exposed to 14 nM RAPA (a specific mTOR inhibitor) in the absence or presence of 144 μM PA for different periods of time (a control for 24 h; b RAPA for 24 h; c PA for 24 h; d PA + RAPA for 24 h; e control for 48 h; f RAPA for 48 h; g PA for 48 h; h PA + RAPA for 48 h) and then stained with Oil Red O. **b** The intracellular lipid content in each group was quantified. **c** Intracellular TG levels were measured. After treatment with 14 nM RAPA or PA as indicated for 24 h or 48 h. **d** Western blotting was used to detect the protein expression levels. **e** qRT-PCR was used to detect the relative mRNA expression levels of lipogenic genes. Data are presented as the means±SDs of three independent experiments, each of which was performed in triplicate (*P < 0.05 versus the control group; #*P* < 0.05 versus the model group)

RAPA is a macrolide immunosuppressant that inhibits the mTOR protein kinase. Thus, using Oil Red O staining and an enzymatic assay kit, we examined whether RAPA is able to prevent sodium palmitate-induced lipid accumulation and increase in TG levels, respectively, in HepG2 cells. To this end, HepG2 cells were exposed to 14 nM RAPA in the absence or presence of a sodium palmitate at a dose of 144 μM for 24 h or 48 h. As shown in Fig. 3a, b and c, RAPA reduced the accumulation of lipid droplets and increase in intracellular TG levels induced by sodium palmitate in a concentration-dependent manner.

To clarify the mechanism of action of RAPA, the protein and mRNA expression levels of lipogenesis-related markers were examined. Treatment with RAPA reduced the increase in the protein expression levels of mTOR, S6K1, LXRα, SREBP-1c, FAS, ACC1 and phosphorylated mTOR, S6K1, and mRNA expression levels of lipogenesis-related markers induced by sodium palmitate (Fig. 3d and e). These inhibitory effects indicated that mTOR is an upstream effector of other genes governing hepatic lipogenesis, such as S6K1, LXRα and SREBP-1c, and SREBP-1c target enzymes.

### Inhibition of S6K1 reduces sodium palmitate-induced lipid accumulation and lipogenesis in HepG2 cells by decreasing the expression of LXRα and lipogenic genes

**Fig. 4:**
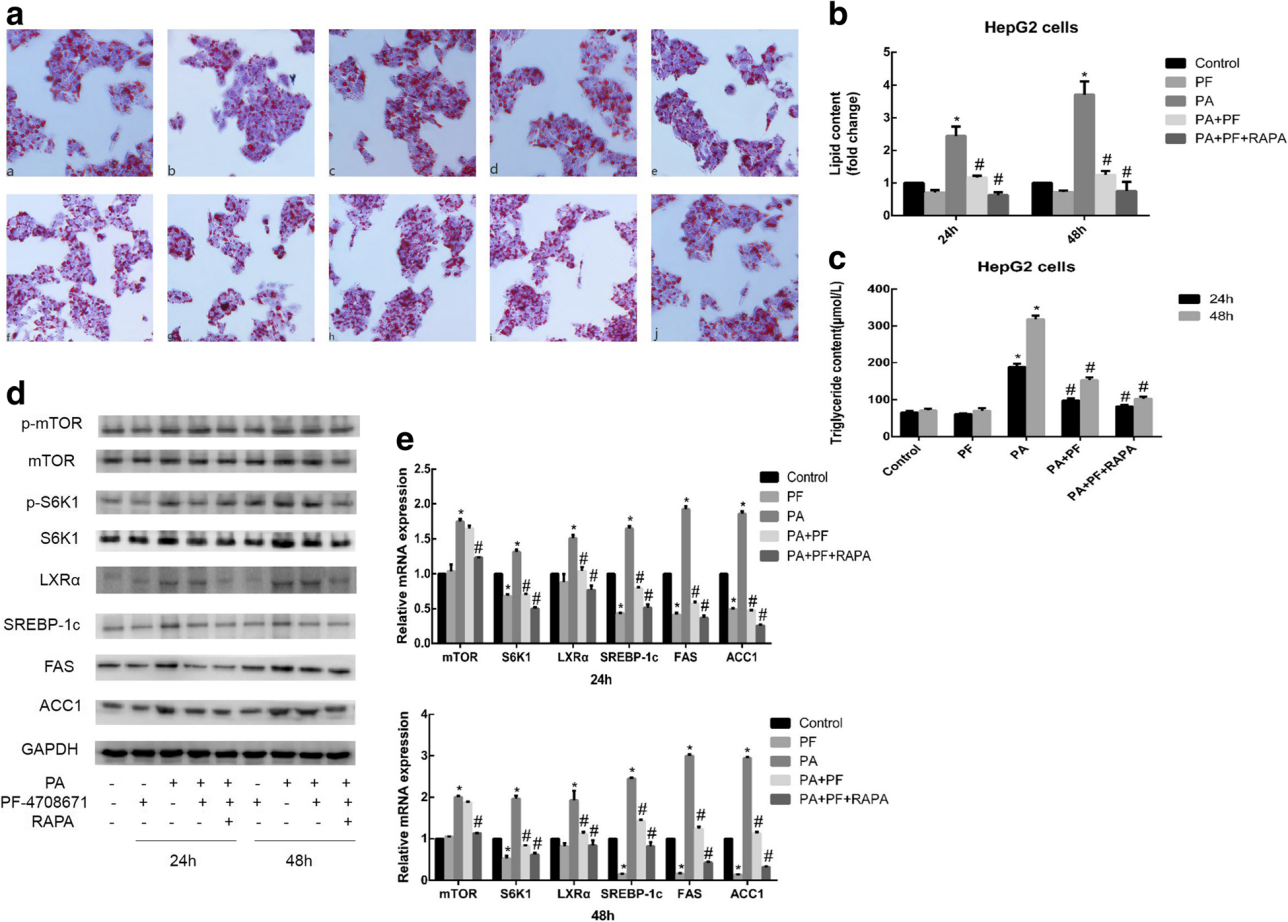
Effect of PF-4708671 on lipid accumulation and expression of lipogenic genes in HepG2 cells. Cells were exposed to 6 μM PF-4708671, 144 μM PA, or 14 nM RAPA for different periods of time. **a** After treatment (a control for 24 h; b PF-4708671 for 24 h; c PA for 24 h; d PA + PF-4708671 for 24 h; e PA + PF-4708671 + RAPA for 24 h; f control for 48 h; g PF-4708671 for 48 h; h PA for 48 h; i PA + PF-4708671 for 48 h; and j PA + PF-4708671 + RAPA for 48 h), the cells were stained with Oil Red O. **b** The intracellular lipid content in each group was quantified. **c** Detection of TG levels. After treatment. **d** Western blotting was used to detect the protein expression levels, and (**e**) qRT-PCR was used to detect the relative mRNA expression levels of lipogenic genes. Data are presented as the means±SDs of three independent experiments, each of which was performed in triplicate (*P < 0.05 versus the control group; #*P* < 0.05 versus the model group)

Using hepatocytes, several studies have shown that downstream of mTORC1, its target S6K1 is phosphorylated at Thr389 in response to PA and that this process regulates subsequent lipogenic gene expression induced by SREBP-1c [18, 19]. To further confirm whether S6K1 is the upstream kinase for SREBP-1c and the downstream effector of mTOR, HepG2 cells were treated with PF-4708671, a novel and highly specific inhibitor of p70 ribosomal S6K1 at the concentration of 6 μM. As shown in Fig. 4a, b and c, PF-4708671 significantly reduced lipid droplet accumulation and increase in intracellular TGs induced by sodium palmitate in HepG2 cells. Moreover, the extent of reduction was enhanced by the combination of PF-4708671 with RAPA. The protein expression levels of S6K1, phosphorylated S6K1, LXRα, SREBP-1c, FAS, ACC1 and the relative mRNA expression levels of lipogenic genes were decreased by PF-4708671 treatment, and the inhibitory effect was further enhanced by combination with RAPA, indicating that SREBP-1c activation and lipogenesis requires the mTOR/S6K1 pathway. However, the expression of mTOR was not affected by PF-4708671 (Fig. 4d and e), which again indicates that S6K1 is a target of mTOR. Interestingly, sodium palmitate-induced increase in LXRα protein and mRNA levels in HepG2 cells were reduced by PF-4708671 treatment, and the extent of reduction was further enhanced by combination with RAPA. Therefore, sodium palmitate induces lipid accumulation via the mTOR/S6K1/SREBP-1c signaling pathway in HepG2 cells, and LXRα may be involved in this process.

### LXRɑ is involved in the mTOR/S6K1/SREBP-1c signaling pathway in sodium palmitate-induced HepG2 cells

**Fig. 5:**
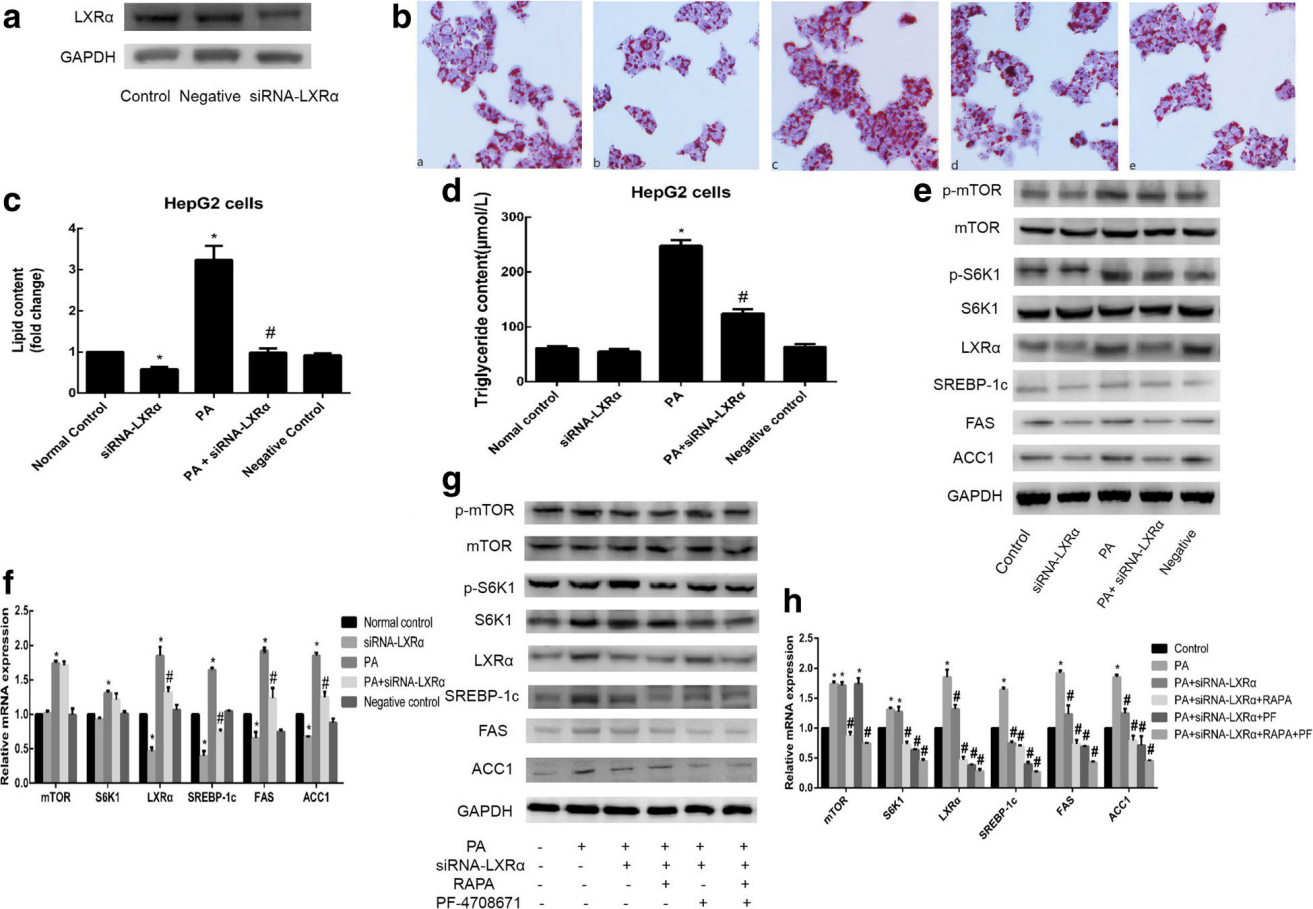
Effect of LXRα knockdown on HepG2 cells. **a** Detection of LXRα knockdown efficiency by Western blotting. **b** Oil Red O staining (a normal control; b siRNA-LXRα; c PA; d PA + siRNA-LXRα; and e negative control). **c** The intracellular lipid content in each group was quantified. **d** Detection of TG levels. After transient transfection of HepG2 cell with siRNA-LXRα in the absence or presence of PA. **e** Western blotting was used to detect the protein expression levels, and (**f**) qRT-PCR was used to detect the relative mRNA expression levels of lipogenic genes. Verification of the inhibitory effect of LXRα knockdown, RAPA, and PF-4708671, alone and in combination. Protein expression levels (**g**) and mRNA expression levels (**h**) were assessed by Western blotting and qRT-PCR, respectively. Data are presented as the means±SDs of three independent experiments, each of which was performed in triplicate (*P < 0.05 versus the control group; #*P* < 0.05 versus the model group)

LXRα is a transcription factor that belongs to the nuclear receptor superfamily and plays a central role in the regulation of cholesterol homeostasis and lipid synthesis. LXRα regulates hepatic lipogenesis mainly by mediating the expression of SREBP-1c and its target genes, such as FAS and ACC1, for FA synthesis. To determine whether LXRα is involved in sodium palmitate-induced lipogenesis in HepG2 cells, we transiently transfected siRNA-LXRα and negative control siRNA into HepG2 cells. Negative control siRNA lacks complementary sequences to the human genome. Indeed, compared to the negative control siRNA, siRNA-LXRα significantly suppressed LXRα protein expression in HepG2 cells (Fig. 5a), indicating that the knockdown of LXRα expression was successful in HepG2 cells. In these LXRα-knockdown HepG2 cells, the accumulation of lipid droplets and the increase in intracellular TG levels induced by sodium palmitate were reduced (Fig. 5b, c and d). Moreover, sodium palmitate-induced increases in LXRα, SREBP-1c, FAS, ACC1 protein and mRNA levels were reversed by siRNA-LXRα transfection, while the expression of mTOR and S6K1 was not notably affected (Fig. 5e and f). Therefore, LXRα is required for sodium palmitate-induced lipogenesis in HepG2 cells, and LXRα is a downstream effector of S6K1.

To further investigate whether S6K1 affects SREBP-1c and its target enzymes via LXRα, we knocked down LXRα in cells and then treated them with sodium palmitate, RAPA, PF-4708671 alone or in combination. As shown in Fig. 5g and h, combination with RAPA or PF-4708671 enhanced the inhibitory effect of LXRα knockdown on LXRα, SREBP-1c, FAS and ACC1 levels. Therefore, sodium palmitate regulates hepatic lipid accumulation through the mTOR/S6K1/LXRα/SREBP-1c signaling pathway in HepG2 cells.

## Discussion

In recent years, the incidence of NAFLD has increased, and NAFLD has emerged as a public health problem. The pathogenesis of NAFLD is well described and involves both genetic susceptibility and the development of a multifactorial metabolic disorders. The persistent intake of a high-fat diet (HFD) is suggested to play a critical role in NAFLD etiology and progression [20]. Hepatic lipid accumulation is observed in various stages of NAFLD, which can ultimately lead to hepatitis and cirrhosis [21]. In the clinic, NAFLD is strongly associated with increased serum FFA levels [22]. Previous studies have provided evidence that saturated FFAs (e.g., PA and stearic acid) are able to induce lipogenesis and lipoapoptosis in human and rat liver cells [23, 24]. HepG2 cells have been frequently used in the literature for establishing in vitro models of fatty liver diseases, e.g., NAFLD [25–27]. In the present study, we demonstrated that sodium palmitate treatment induced lipid accumulation and upregulation of lipogenic gene expression in HepG2 cells. This study corroborated previous findings and further provided insights into a molecular signaling pathway in the sodium palmitate-treated cells.

The mTOR signaling pathway is involved in many of the major cellular functions, with important roles in regulating protein synthesis, cell cycle progression and proliferation [5]. Moreover, mTOR is an important factor in the occurrence and development of NAFLD, as it is involved in the induction of insulin resistance and chronic hepatitis [28, 29]. It has been reported that the expression of SREBP-1c induced by insulin in cultured hepatocytes is sensitive to RAPA, suggesting that mTOR is responsible for regulating SREBP-1c expression [12]. SREBP-1c is the primary transcription factor that regulates the transcription of genes involved in lipid and FA synthesis, and it is regulated by glucose and insulin levels [30]. Moreover, S6K1 is a downstream effector of mTORC1, and the regulation of SREBP-1c expression has been reported to be both independent of and dependent on S6K1 levels [9, 12]. Thus, the role of the mTOR signaling pathway in the regulation of SREBP-1c expression in the liver has remained unclear.

To assess the role of the mTOR signaling pathway in the occurrence and development of NAFLD, we examined whether treatment with RAPA and PF-4708671 (specific inhibitors of mTOR and S6K1, respectively) can inhibit intracellular lipid accumulation in HepG2 cells. Both RAPA and PF-4708671 could independently decrease the accumulation of TGs and lipid droplets in sodium palmitate-induced HepG2 cells, and the combined inhibitory effect of these two agents was even more apparent. Next, we showed that the mTOR signaling pathway activated by sodium palmitate promotes the expression of genes encoding mTOR, S6K1, LXRα, SREBP-1c, FAS and ACC1 in cultured cells. This increase in gene expression was completely inhibited by RAPA and partially inhibited by PF-4708671, which suppressed all genes except for the mTOR gene, indicating that S6K1 is responsible for the mTOR-mediated regulation of expression of SREBP-1c and its target lipogenic genes (FAS and ACC1) and that LXRα may be a downstream of mTOR or S6K1.

LXRs are ligand-activated nuclear receptors belonging to the steroid hormone receptor superfamily that act as oxysterol sensors and can regulate genes involved in cholesterol transport, lipid metabolism, and carbohydrate metabolism [31]. There are two LXR isoforms–the ɑ isoform is expressed most highly in the liver and to a lesser extent in the kidney, small intestine, spleen, and adrenal glands, whereas the β isoform is ubiquitously expressed [32–34]. Moreover, the ɑ isoform is the predominant isoform that functions as a master hepatic lipogenic transcription factor. Previous studies have shown that genes involved in hepatic FA synthesis, such as SREBP-1c, FAS and ACC1, are downregulated in LXRɑ-deficient mice and that the expression of the above genes remains unaffected in LXRβ-deficient mice [35, 36]. The current study demonstrated that LXRα controls hepatic lipogenesis primarily by mediating the expression of SREBP-1c and its target genes, such as FAS and ACC1, in FA synthesis [37]. In our study, sodium palmitate stimulated the expression of LXRα, and siRNA-mediated depletion of LXRα suppressed the expression of SREBP-1c and its target genes (FAS and ACC1), in accordance with the results of previous reports.

It has been previously shown that S6K1 directly phosphorylates LXRα at serine residues to promote gene transactivation [13]. Thus, we investigated whether there is a cross-talk between LXRα and the mTOR/S6K1/SREBP-1c signaling pathway. In our study, RAPA and PF-4708671 reduced the sodium palmitate-induced increase in the expression of LXRα, and the combined inhibitory effect of these two agents was even more apparent.However, LXRα knockdown had no inhibitory effect on mTOR or S6K1 expression. In addition, RAPA and PF-4708671 could enhance the inhibitory effect of LXRα knockdown. Hence, LXRα may be a downstream effector of S6K1 in the mTOR signaling pathway.

## Conclusions

In conclusion, sodium palmitate regulates hepatic lipid accumulation by modulating the mTOR/S6K1/LXRα/SREBP-1c signaling pathway. These results broaden our understanding of NAFLD pathogenesis. NAFLD represents the hepatic manifestation of metabolic syndromes, and understanding NAFLD pathogenesis is important for developing novel therapeutic interventions. To date, the mTORC1 inhibitor RAPA has largely been used to reduce the expression of SREBP-1c, a key lipogenic transcription factor. However, this is not always ideal, as individuals treated with RAPA or a RAPA analogs have been found to experience an increase in TG levels. The reason may be that mTORC1 also phosphorylates other substrates such as the autophagy kinase ULK1, which can accelerate lipid accumulation. In the present study, for the first time, we described the mTOR/S6K1/LXRα/SREBP-1c signaling pathway. Our findings suggest that LXRα might be a potential therapeutic target for NAFLD.

## Abbreviations

ACC-1: Acetyl-CoA carboxylase-1
ER: Endoplasmic reticulum
FAS: Fatty acid synthase
FAs: Fatty acids
FFAs: Saturated free fatty acids
LXRα: Liver X receptor-α
mTOR: The mammalian target of rapamycin
NAFLD: Non-alcoholic fatty liver disease
PA: Sodium palmitate
RAPA: Rapamycin
S6K1: S6 kinase 1
siRNA: Small interfering RNA
SREBP-1c: Sterol regulatory element-binding protein-1c
TC: Cholesterol
TGs: Triglycerides
